# Morphological Profiles of RNAi-Induced Gene Knockdown Are Highly Reproducible but Dominated by Seed Effects

**DOI:** 10.1371/journal.pone.0131370

**Published:** 2015-07-21

**Authors:** Shantanu Singh, Xiaoyun Wu, Vebjorn Ljosa, Mark-Anthony Bray, Federica Piccioni, David E. Root, John G. Doench, Jesse S. Boehm, Anne E. Carpenter

**Affiliations:** Broad Institute of Harvard and MIT, Cambridge, Massachusetts, United States of America; Baylor College of Medicine, UNITED STATES

## Abstract

RNA interference and morphological profiling—the measurement of thousands of phenotypes from individual cells by microscopy and image analysis—are a potentially powerful combination. We show that morphological profiles of RNAi-induced knockdown using the Cell Painting assay are in fact highly sensitive and reproducible. However, we find that the magnitude and prevalence of off-target effects via the RNAi seed-based mechanism make morphological profiles of RNAi reagents targeting the same gene look no more similar than reagents targeting different genes. Pairs of RNAi reagents that share the same seed sequence produce image-based profiles that are much more similar to each other than profiles from pairs designed to target the same gene, a phenomenon previously observed in small-scale gene-expression profiling experiments. Various strategies have been used to enrich on-target versus off-target effects in the context of RNAi screening where a narrow set of phenotypes are measured, mostly based on comparing multiple sequences targeting the same gene; however, new approaches will be needed to make RNAi morphological profiling (that is, comparing multi-dimensional phenotypes) viable. We have shared our raw data and computational pipelines to facilitate research.

## Introduction

The systematic perturbation of genes by RNA interference has been used to identify novel players in many biological processes and pathways [1–3]. Although off-target effects of RNA interference reagents are known to complicate such experiments [4–6], many findings have been thoroughly validated. High-throughput microscopy is frequently a readout of choice for large-scale screens, including those involving RNAi [7–9]. Usually, only one or two morphological features of cells are measured from images in order to score samples [10]. However, much more information can be extracted from images, making it a “high-content” data source. Multiple stains can be employed to visualize a range of cellular components, enabling hundreds of cellular morphology features to be measured at a single-cell level in a single assay.

Imaging is thus a potentially powerful candidate readout for ‘profiling’–defined as the systematic harvesting of hundreds or thousands of distinct measurements in parallel, and the subsequent mining of these large arrays of measurements, termed profiles, for similarities and patterns. These similarities and patterns can allow powerful data-driven analyses, for example, to identify signatures associated with disease, connect disease targets with potential therapeutics, assess the impact of genetic mutations, and identify compounds with similar function to query compounds of interest [11–25].

The goals and practice of profiling are quite different from screening for a narrow, pre-defined set of phenotypes. In high-content screening based on lower-dimensionality readouts, the phenotypes of interest (e.g., cell ploidy, staining intensity, a particular morphology) have already been identified and controls are usually available. Even if pattern recognition tools are needed to score for the phenotypes based on multiple cell measurements [26–31], the aim is still to select a small number of hits based on the narrow, predefined set of phenotypes. By contrast, the goal of profiling is to look for patterns across the entire dataset based on a wide spectrum of cellular features, typically hundreds. Positive and negative controls are often difficult or impractical to define.

Image-based profiling has been successful using small molecules as the perturbing reagents [8,12–16,18,25,32]. In our own experience, we have used image-based profiling to classify the mechanism of action of compounds [33], to cluster compounds into meaningful groups [34], and to create a performance-diverse compound library [35].

However, in contrast to the multitude of image-based screening experiments using RNAi, there are few published studies of image-based profiling that characterize cells perturbed by RNAi [19–22,32]. Why is this the case? One possible explanation is simply that image-based profiling is in its infancy: even for experiments involving small molecules, there are only a dozen published experiments.

Here, we explore an additional hypothesis, that RNAi image-based profiling has not been widely successful because the phenotypic effects resulting from reducing the expression of the target gene (“on-target effects”) are too obscured by changes induced by reducing the expression of unrelated genes (“off-target effects”) to identify the on-target effects. In particular, we suspected that seed-sequence effects, which result from the binding of a short “seed” region of RNAi reagents to multiple messenger RNAs and are known to complicate screening experiments [6,36–40], might preclude similarity matching in high-dimensional profiling experiments. In one study using gene expression profiles as a high-dimensionality readout, significant off-target effects mediated by seed sequences were identified for a handful of RNAi targets [41].

We find that for image-based profiling of a larger number of gene targets, pairs of RNAi reagents that share the same seed sequence produce morphological profiles that are much more similar to each other than profiles from pairs designed to target the same gene. We note that our experiment involves broad image-based profiling of shRNA effects in mammalian cells. It may be that in other systems, such as long dsRNA libraries in *Drosophila* cells, the relative magnitude of seed-driven effects might be smaller as evidenced by higher concordance of distinct reagents targeting the same gene [42]. Still, given the history of off-target effects in all systems, including *Drosophila* [43–45], carefully designed controls and validation are warranted.

Overall, our finding indicates that for RNAi to be successful for profiling, advances in data interpretation are needed beyond what has been used for simpler types of RNAi screen data.

## Results

### An RNAi image-based profiling assay

**Fig 1:**
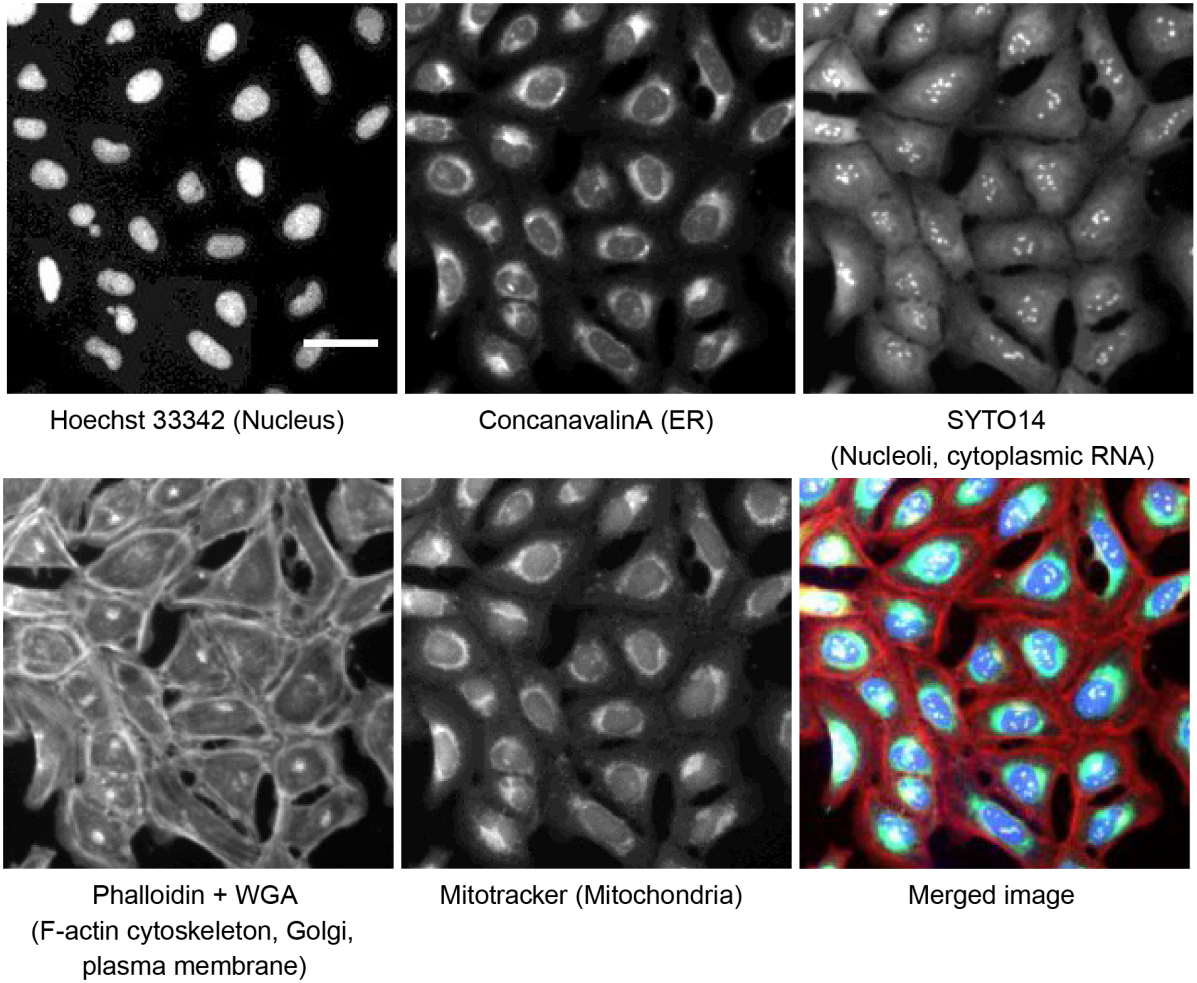
Cell Painting assay. U2OS cells prepared for this study were stained using the Cell Painting assay protocol [34], with six stains imaged across five channels, revealing eight cellular components/structures. Scale bar 25 μm.

We devised a profiling experiment whose ultimate aim was to place uncharacterized genes into pathways based on similarities to known genes, in terms of morphological phenotype. As an initial test of whether RNAi could yield sensitive and reproducible image-based profiles, we used a microscopy assay we previously developed called “Cell Painting” [34] (Fig 1). Its multiple stains provide a snapshot of the morphology of eight cellular structures/components; the assay has been used thus far to group compounds by similarity [34] and to profile a large compound library [35].

**Fig 2:**
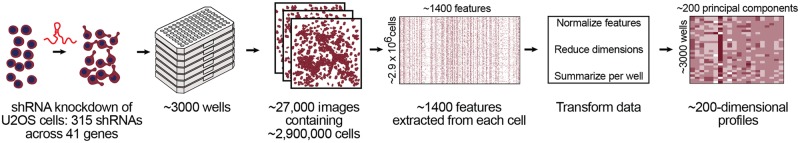
Workflow for generating morphological profiles using Cell Painting. U2OS cells are treated with shRNAs and transferred to 384-well plates in which they are stained and then imaged. The images are analyzed and ~1400 features are extracted from each cell. These data are then transformed to generate multivariate profiles.

We targeted a set of 41 genes in the U2OS cell line using short-hairpin RNAs (shRNAs) in arrayed, 384-well format (see Materials and Methods). Most genes were targeted by at least six distinct shRNAs and the experiment was performed in eight replicates. Using our open-source CellProfiler software [46], we identified cellular compartments and structures across the different channels and extracted 1402 morphological features for every cell imaged in the experiment (Fig 2). Features include metrics such as staining intensities, size, shape, and texture of the various cellular structures, as well as correlation between stains across channels and neighborhood relationships among cells and among intracellular structures. From this raw per-cell data, we constructed morphological profiles for each shRNA reagent (see Materials and Methods).

### Different shRNAs targeting the same gene have distinct profiles

**Fig 3:**
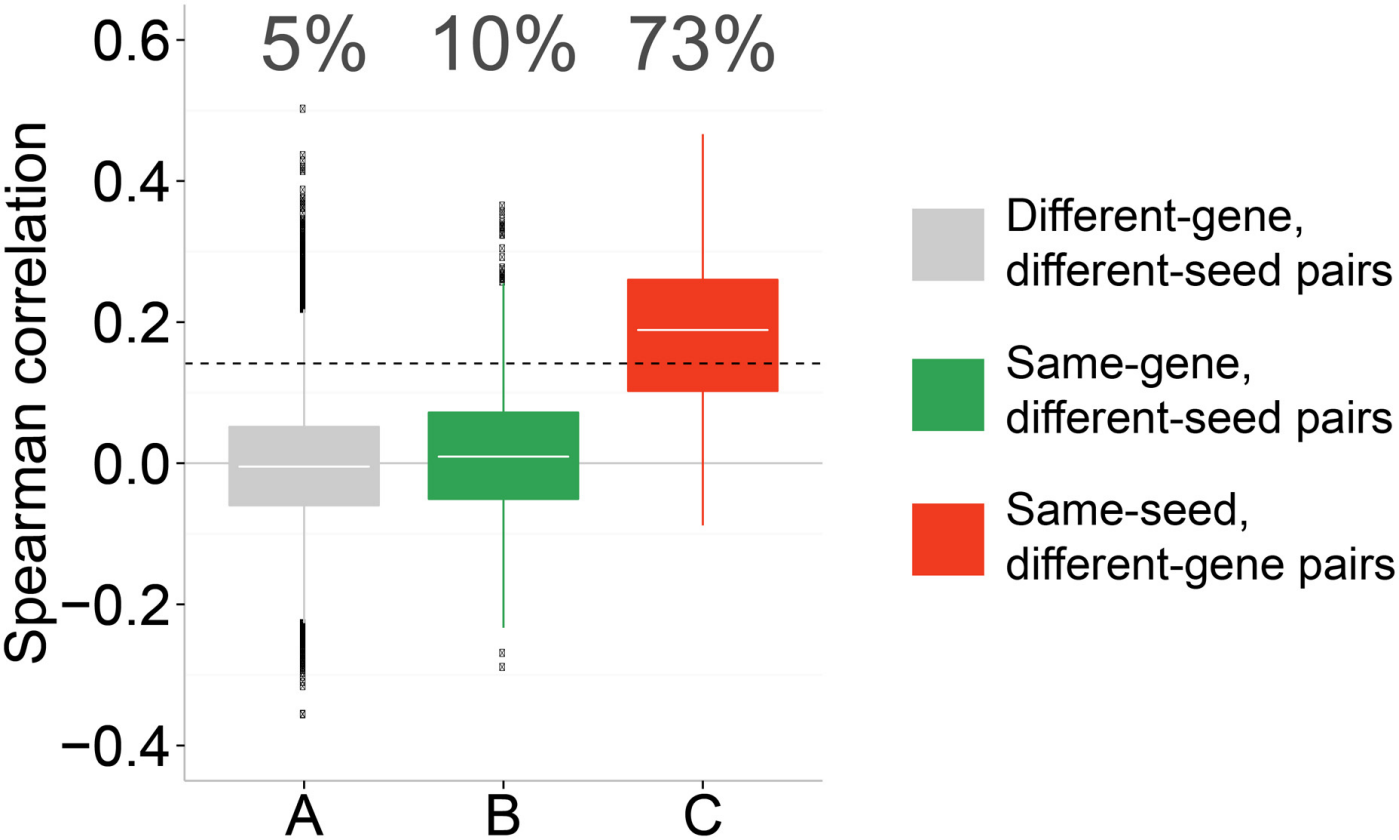
Different RNAi reagents targeting the same gene rarely produce similar profiles, whereas RNAi reagents sharing a seed sequence do. shRNAs targeting the same gene have very low correlation (B), whereas those containing the same seed sequence have a much higher correlation (C). Using the 95^th^ percentile of a null distribution (A) as a threshold to define significant correlations, only 10% of correlations in B are significant, compared to 73% in C. This indicates that the phenotypes induced by RNAi knockdown are dominated by seed effects. Correlations are computed between profiles of sequences, obtained by median-averaging profiles of replicate wells. The percentage of correlations above the defined threshold is indicated for each group; dotted line indicates 95^th^ percentile of the null distribution (A). The difference between means of B and C is highly significant (*P*-value < 10^−5^; two-sided Student's *t*-test).

We tested how frequently different shRNA sequences targeting the same gene have similar morphological profiles. Although we anticipated that off-target effects would make many shRNAs that target the same gene look dissimilar, we were surprised to find that nearly all same-gene shRNAs were dissimilar from each other. In fact, the correlations between profiles of shRNAs targeting the same gene are comparable in magnitude to the correlations between shRNAs targeting different genes: both average around zero (Fig 3A and 3B), consistent with similar observations in a previous study using lower-dimensional image-based profiles focused on endocytosis [21] as well as high-throughput gene-expression data (our unpublished results). This implies that either the Cell Painting assay is not sensitive enough to create reproducible profiles, or that it is, but that the same-gene shRNAs induce very different morphological phenotypes in cells.

### Image-based profiles induced by RNAi are highly reproducible

**Fig 4:**
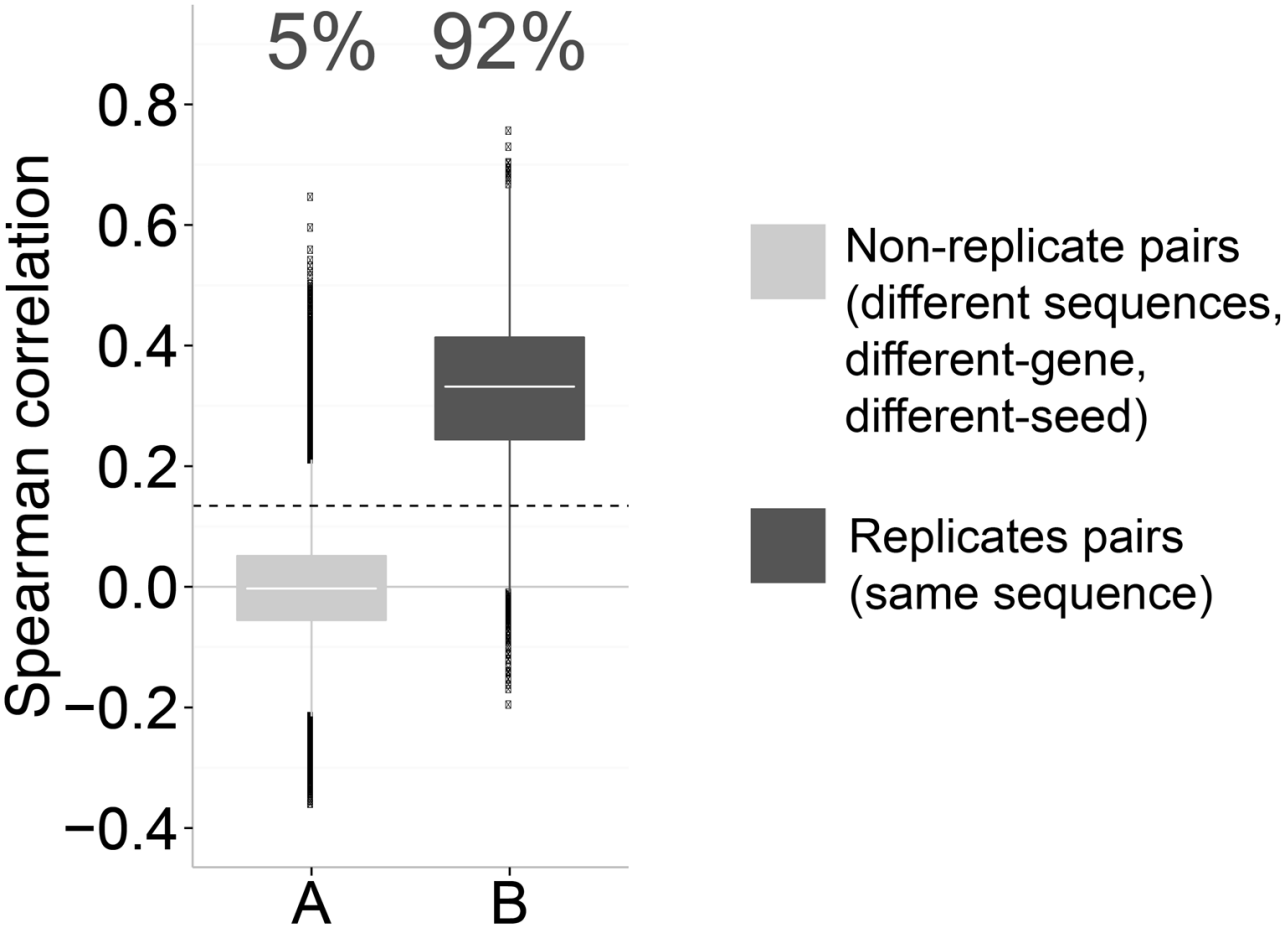
Image-based profiles of RNAi sequences are highly reproducible. Using the 95^th^ percentile of the null distribution (A) as a threshold to define significant correlations, 92% of replicate correlations in B are seen to be significant. Correlations are computed between profiles of individual wells. The percentage of correlations above the defined threshold is indicated; dotted line indicates 95^th^ percentile of the null distribution (A). The difference between means of A and B is highly significant (*P*-value < 10^−15^; two-sided Student's *t*-test).

We next observed that more than 90% of shRNA replicate pairs—repeat measurements employing the same shRNA—were significantly correlated (Fig 4); the result holds true even when replicates were constrained to come from different well positions on the plate (S1 Fig). Therefore, the morphological effect induced by an individual shRNA sequence, and thus its profile, is highly reproducible. We draw two conclusions: (1) the morphological phenotypes induced by nearly all shRNAs are distinguishable from each other, and (2) image-based profiles induced by RNAi are highly reproducible. Together with our initial result we conclude that shRNAs meant to target the same gene often induce very different morphological phenotypes in cells. We observe that this result holds true even with putative negative control shRNAs, that is, shRNA sequences not matching any genes in the cell’s genome (S2 and S3 Figs). We find that more than 90% of shRNA replicate pairs targeting these so-called negative controls are significantly correlated (S2 Fig) and that different shRNA sequences targeting the same negative control (unexpressed) gene look dissimilar (S3 Fig).

### The off-target seed effect is stronger than the on-target effect

Although different morphological effects might result from shRNAs knocking down target genes to different levels, we first investigated whether known off-target effects might be dominating the morphological profiles. RNAi reagents are involved in at least two pathways: the RNAi pathway and the microRNA pathway [47,48]. In the canonical RNAi pathway, 18–22 nucleotides of sequence similarity are needed to direct the degradation of a target mRNA; this is the pathway responsible for on-target effects. In the microRNA pathway, a specific 6–8 nucleotide portion of the shRNA, the “seed sequence,” plays a key role in target recognition. Its shorter length results in lower specificity––potentially repressing many hundreds of messenger RNAs and contributing to the off-target effects of the shRNA or siRNA [41,47,49] that complicate interpretation of screening data [37–40].

**Fig 5:**
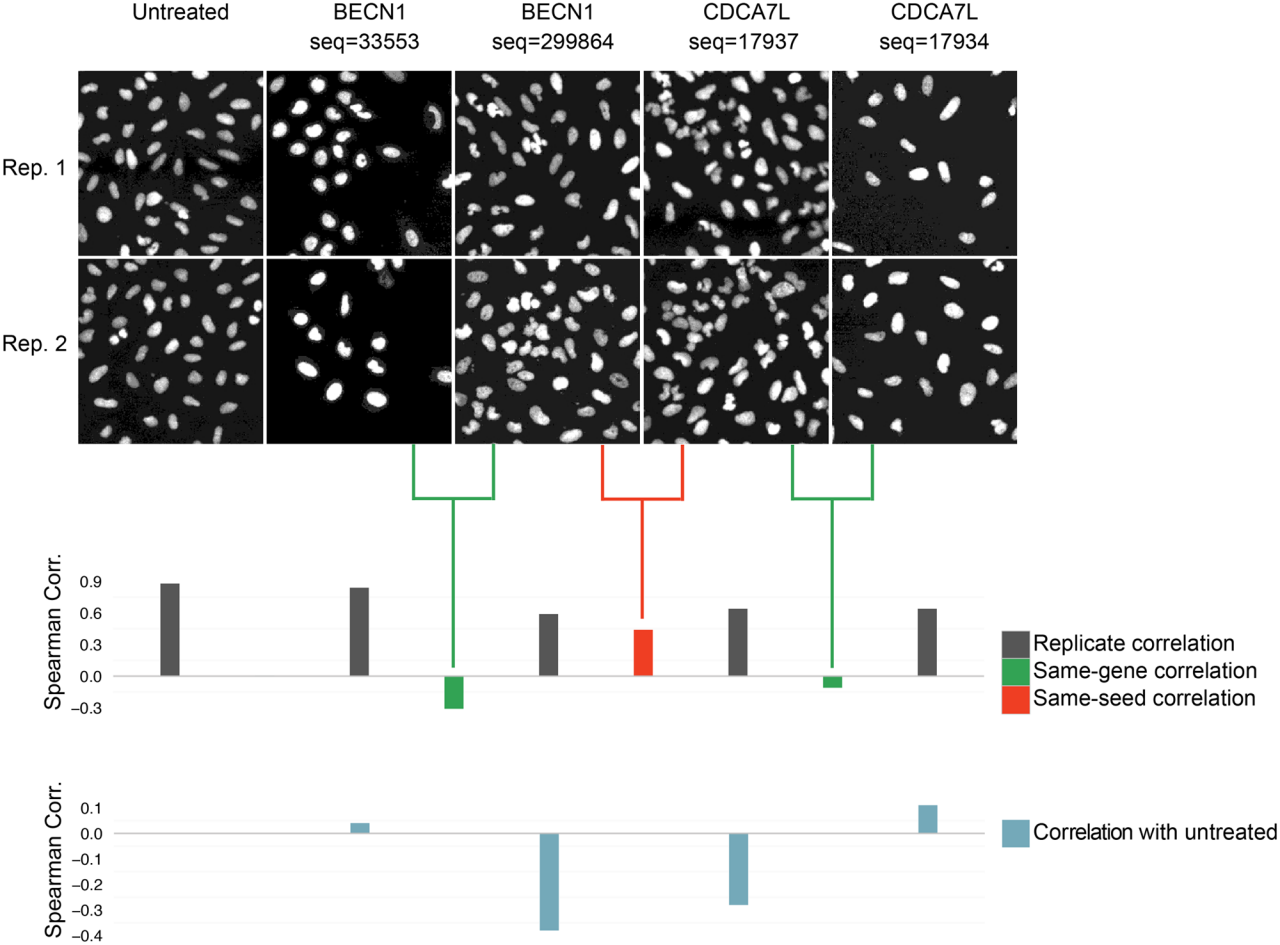
Visual example of seed effects dominating morphological profiles. Only nuclear shape features were used in this example in order to yield visually interpretable phenotypes. Two of the shRNA sequences targeting CDCA7L and BECN1 share a seed sequence (sequences 299864 and 17937 have a common seed GAATGA at nucleotides 12–17). The Spearman correlation between the morphological profiles of these shRNAs is high (seed correlation = 0.44, red bar). For each of these two shRNAs, a same-gene shRNA with a dissimilar phenotype is also shown (same-gene shRNA correlation = -0.31 and -0.11, green bars). All four shRNAs have high replicate correlation and are dissimilar from untreated cells. We specifically chose an example where the seed correlation is high and the same-gene shRNA correlations are low; however this phenomenon is seen globally (Fig 3). Images have been zoomed in, showing only 2% of the imaged region.

We found that pairs of shRNAs that share the same seed sequence produce image-based profiles that tend to be more similar to each other than profiles from shRNAs designed to target the same gene (Fig 3C). In fact, more than 70% of the seed pairs had significantly correlated profiles, compared to only 10% of same-gene pairs. This implies that the off-target effect from the microRNA pathway—the so-called *seed effect*—typically dominates the morphological profiles. A visually interpretable example of this phenomenon is illustrated in Fig 5. The high correlation between same-seed shRNAs also rules out more trivial explanations for the low correlation between same-gene shRNAs, such as a poor assay or computational methodology, which in any event have been validated in other contexts [34,35]. It is worth noting that same-gene pairs do indeed have more high-correlation instances than random or mismatched shRNA pairs (10% vs. 5% of instances, respectively, exceed 95% of the null distribution; *P*-value < 10^−5^ for two-sided Student's *t*-test), and these may indeed reflect significant on-target-driven morphology effects. However, given the much higher prevalence of strong seed-driven effects on the overall morphological signature, these cases would be quite difficult to identify and validate.

## Discussion

Our observations indicate that RNAi can induce remarkably strong, detectable, and reproducible morphological effects but that off-target ‘seed’ effects dominate the profiles. While the presence of seed-sequence—driven off-target effects is not surprising, as they were discovered more than a decade ago [3,41,48], the magnitude of the problem had not previously been quantified across multiple genes in the context of high-dimensional RNAi profiling, particularly when using imaging.

We found that the magnitude and prevalence of off-target effects is powerful enough that, most of the time, morphological profiles of RNAi reagents targeting the same gene look no more similar than reagents targeting different genes. We do not think this is unique to imaging profiles, as we have seen poor correlations in high-throughput gene expression profiles as well (our unpublished results). This indicates that morphological profiles based on RNAi cannot be used as a reliable readout of a particular gene’s impact without new methods to extract the on-target component of the profile from the rest of the signal (discussed later).

If RNAi reagents generally produce diverse, off-target morphological phenotypes so strong as to confound profiling experiments, one could wonder how RNAi high-content screens have yielded useful results. First, it should be noted that the results of RNAi screens often do *not* substantially overlap; meta-analyses of RNAi screens have indicated this [6,50]. Specifically, a recent study on a set of infection screens has suggested that a majority of hits could be attributed to the seed effect [6].

Still, screening for a narrow pre-defined set of phenotypes may have enjoyed greater success due to fundamental differences versus profiling. Generally, off-target effects are confounding only if they induce a detectable phenotype in the assay being employed. For a set of narrowly defined phenotypes of interest, the probability of any particular off-target mechanism inducing those particular phenotypes is relatively low (roughly equivalent to the hit rate of the screen, often 0.1–5%). Thus, approaches to identify and then exclude or de-emphasize shRNAs that are acting on the assayed phenotypes through the seed effect have been shown to improve screening results [37,39]. In the case of profiling, however, so many features are measured that it is much more likely that there are at least some that will be sensitive to the off-target effect of a given RNAi sequence.

RNAi has also likely been more successful in the context of screening for more narrowly defined phenotypes because the goal is only to choose a small number of hits at the top of a rank-ordered list for each specific phenotype of interest. As RNAi screening experts have long stressed [5,51,52], multiple hairpins per gene should be analyzed; a number of methods have been introduced to select hits from multiple-hairpin data to reduce the chances of the phenotype being a result of an off-target effect, as summarized recently [53]. However, in profiling experiments, off-target effects are much more likely to be confounding because the goal is to compare the multi-dimensional patterns produced by *all* the reagents, not just a tiny subset at the very top of a list.

Several workarounds may be worth pursuing to make RNAi viable for profiling. Any such solution must accommodate that *all* RNAi reagents, including putative negative controls, have a seed sequence and seed activity. At present, all reagents with siRNA activity also additionally produce this seed effect, such that it is not possible to simply pre-filter libraries of RNAi reagents (e.g. shRNAs or siRNAs) that avoid any seed effect. While a particular seed may appear ‘silent’ in a specific context (i.e. a specific cell line and assay readout) and this can empirically mitigate off-target concerns for that reagent *within that particular context*, this does not guarantee that it will not produce a detectable effect in any new context. Chemical modifications to the guide and passenger strands is a strategy introduced in many commercial siRNA libraries [54], though this can only reduce but not eliminate seed effects. Another strategy is to use sequence-specific controls [36] for each RNAi reagent to help factor out off-target effects, but these are yet to be widely available. Delivering multiple independent RNAi reagents targeting each assayed gene in every screened cell has been proposed as a means to dilute seed effects while preserving on-target activity [48,55,56]. Small pools of a few reagents per gene may not be so helpful in this regard since this increases the number of seeds, and the activity of each seed contributor may not be sufficiently diluted in magnitude-of-effect to offset this disadvantage. It has been claimed that much larger pools of dozens of siRNAs may, on balance, reduce off-target effects [47,52]. The technology remains to be scaled up, independently validated, and tested in the context of profiling, and has not come into widespread usage.

When appropriate to the experimental design, one simple solution to capturing gene function information is to switch to a gain-of-function rather than loss-of-function strategy, that is to overexpress genes rather than suppressing their expression [57]; we are currently following this path in several experiments. If reducing gene expression is preferred or required, CRISPR [58] and related technologies may accomplish this with fewer off-target effects, although the consequences to an extensive profiling-type readout, tested across many perturbations and targets, have yet to be reported.

Finally, some groups have worked on computational solutions to handle off-target effects for profiling applications. For example, one approach is to retain only those genes for which the profiles of same-gene RNAi reagent pairs “match” each other [20]. This approach was tested in an experiment with 11 image-based features that yielded strong matches between same-gene RNAi pairs for 87% of genes. Our results indicate that this strategy is unfortunately not extendable to higher-dimensional profiles, such as those presented here, for which very few same-gene pairs of profiles show any similarity. It has also been proposed to use orthogonal information (e.g., protein-protein interaction data) to inform the creation of profiles [23] but this has not been demonstrated to work at the level of individual genes, as opposed to gene families, nor to overcome seed effects. Another approach is to use a probabilistic model to “average” the profiles of same-gene RNAi reagents to create a gene-specific signature [21], but the method has neither been published nor demonstrated in subsequent experiments.

In summary, none of the proposed approaches has been proven to resolve the seed effect problem—a source shown here to dominate off-target effects in RNAi—in particular by testing whether the seed effect is indeed absent in the deconvoluted profiles. Given that most of the existing approaches have been tested on ~10–50-dimensional profiles, it is likely that applying them to profiles with hundreds to thousands of dimensions would be expected to work less well, for the same reasons that screening for a narrow, pre-defined phenotype has a more straightforward path to identifying and disentangling off-target effects than profiling. Additionally, solutions may need to be modality-specific because the assumptions made about the structure of the data, such as additivity of features or correlations, that are valid for one modality may not be valid for the other (e.g. gene expression vs. morphological profiling). Finally, developing computational approaches to solve the seed effect problem for high-dimensional profiling experiments would require conducting large experiments with multiple RNAi reagents targeting each gene, as well as designing multiple RNAi reagents with the same seed sequence.

We have provided our raw data and computational pipelines to facilitate further research.

## Materials and Methods

### Selection of genes

We selected 41 genes based on multiple criteria. Expression of the gene in the U2OS cell line was required, which was verified using existing Affymetrix data. Genes with known biological function or those that are known to produce morphological phenotypes upon knockdown were preferred. shRNAs were obtained from The RNAi Consortium (TRC) Lentiviral shRNA Library (http://www.broadinstitute.org/rnai/public/).

### Selection of shRNAs

shRNAs were selected to span a range of knockdown efficiencies to avoid the dataset being biased towards high knockdown efficiency alone. Knockdown efficiency was observed not to correlate with morphological profile robustness, measured as the median of the Spearman correlation between all its replicates (data not shown). In all, 37 out of the 41 genes had six or more shRNAs targeting them. A small number of shRNAs were excluded after being filtered out in the image processing quality control step. The 315 unique shRNA sequences used in the analysis are listed in S5 Dataset.

### Quantification of shRNA knockdown efficiency by qPCR

Relative gene expression—the percentage of target transcript remaining after knockdown of the target gene by each construct—was quantified (S8 Dataset) using the ddCt method, described in detail in Section 15.8.9 of Bookout et al. [59].

### Cell culture

U2OS cells (#HTB-96, ATCC) were plated at the density of 200 cells per well in 384-well imager quality black/clear plates (Aurora Biotechnologies/Nexus Biosystems). Cells were infected with an arrayed set of shRNA lentiviruses. Six replicates of each plate layout (see below) were prepared. Two replicates were used to assess infection efficiency, with puromycin added to one replicate and the other replicate left untreated. Viability for these two replicates was quantified 96 hours post-infection using Cell Titer-Glo. The infection efficiency was determined by comparing luminescence values of puromycin-treated cells to luminescence values of non-treated cells and expressed as a percentage (S7 Dataset, Column “IE”). The remaining four replicates were processed for Cell Painting assay at 96 hours post-infection. The experiment was carried out using two plate layouts in order to have shRNAs in different positions, resulting in eight Cell Painting replicates in total.

### Cell staining and imaging

The assay followed a previously published protocol [34]. Briefly, eight different cell compartments and organelles were stained with fluorescent dyes: nucleus (Hoechst 33342), endoplasmic reticulum (concanavalin A/AlexaFluor488 conjugate), nucleoli and cytoplasmic RNA (SYTO14 green fluorescent nucleic acid stain), Golgi apparatus and plasma membrane (wheat germ agglutinin/AlexaFluor594 conjugate, WGA), F-actin (phalloidin/AlexaFluor594 conjugate) and mitochondria (MitoTracker Deep Red). WGA and MitoTracker were added to living cells, with the remaining stains were carried out after cell fixation with 16% paraformaldehyde. Images from five fluorescent channels were captured at 20x magnification on an ImageXpress Micro epifluorescent microscope (Molecular Devices): DAPI (387/447 nm), GFP (472/520 nm), Cy3 (531/593 nm), Texas Red (562/642 nm), Cy5 (628/692 nm). Nine sites per well were acquired, with laser based autofocus using the DAPI channel at the first site of each well.

### Image processing and feature extraction

CellProfiler [46] software version 2.1.0 was used to identify and segment cells and measure cellular features. We used pipelines described and provided by Gustafsdottir et al. [34] (S1 Dataset) to correct for uneven illumination and segment cells into nuclei, cell body and cytoplasmic sub-compartments. 69 wells containing blurry images were excluded, retaining 3003 wells in the experiment. Morphological, intensity, textural and adjacency statistics were measured for each sub-compartment [34]. The 1402 cellular features thus extracted were normalized as follows: For each feature, the median and median absolute deviation were calculated across all untreated cells within a plate; feature values for all the cells in the plate were then normalized by subtracting the median and dividing by the median absolute deviation (MAD) times 1.4826 [60]. Features having MAD = 0 in any plate were excluded, retaining 1301 features in all.

### Creating per-well and per-sequence profiles

Multivariate profiles for each well were computed as follows. First, for each of the 3003 wells, the median for each feature was computed across the cells in the well, resulting in a 1301-dimensional profile per well. Principal components analysis (PCA) was used to reduce the dimensionality of the data, retaining 99% of the variance, resulting in a 205-dimensional feature space. This 205-dimensional vector is the multivariate profile used in the analysis. The per-sequence profile was obtained by computing the median of all the replicates of that sequence, where the median is computed for each feature across all the replicates.

### Reproducibility

We provide (S1 Text) the complete image set, the CellProfiler pipelines used to identify and measure the cells, the dataset of morphological profiles, and the source code for the programs that analyze the profiles and produce the figures in this article.

## Supporting Information

S1 Dataset CellProfiler pipelines. (ZIP)

S2 Dataset CellProfiler illumination functions. (ZIP)

S3 Dataset Image features extracted by CellProfiler. (ZIP)

S4 Dataset Source code for analyzing image features. (ZIP)

S5 Dataset The 315 shRNA sequences profiled using our assay. (ZIP)

S6 Dataset Image feature names measured for each cell by CellProfiler (see the CellProfiler manual for descriptions of each feature). (ZIP)

S7 Dataset Experimental metadata. (ZIP)

S8 Dataset Quantification of shRNA knockdown efficiency by qPCR. Note that this information is based on single-biological replicate HT qPCR measurements in various cell lines, but not U2OS cells. Nevertheless, this should provide a good indication of relative shRNA on-target efficacy among the shRNAs for each gene.
(ZIP)

S1 Fig Image-based profiles of RNAi sequences are highly reproducible, even across different well positions. Treatment replicates coming from different well positions (across plates with different layouts) were compared. Using the 95^th^ percentile of the null distribution (A) as a threshold to define significant correlations, 92% of the replicate correlations in B are seen to be significant. Correlations are computed between profiles of individual wells. The percentage of correlations above the defined threshold is indicated; dotted line indicates 95^th^ percentile of the null distribution (A). The difference between means of A and B is highly significant (*P*-value < 10^−15^; two-sided Student's *t*-test).
(TIF)

S2 Fig Image-based profiles of RNAi sequences are highly reproducible, even for putative negative controls. RNAi reagents with sequences not matching any genes in the cell’s genome—thereby putative negative controls—were analyzed for the reproducibility of their image-based profiles. Specifically, shRNA sequences against GFP, LacZ, Luciferase and RFP were considered. 93% of the replicate pairs of these treatments were significantly correlated. Correlations are computed between profiles of individual wells. The percentage of correlations above the defined threshold is indicated; dotted line indicates 95^th^ percentile of the null distribution (A). The difference between means of A and B is highly significant (*P*-value < 10^−15^; two-sided Student's *t*-test).
(TIF)

S3 Fig Different RNAi reagents targeting the same gene rarely produce similar profiles, even for putative negative controls. For the putative negative controls considered in S2 Fig, shRNAs sequences targeting the same (non-existent) genes had very low correlation (B). Data was insufficient to do a seed analysis similar to Fig 3C; however the off-target effect is likely to be due to the seed effect. Correlations are computed between profiles of sequences, obtained by median-averaging profiles of replicate wells. The percentage of correlations above the defined threshold is indicated; dotted line indicates 95^th^ percentile of the null distribution (A).
(TIF)

S1 Text Data and software. (DOC)
