# Supplementary figures and images for: Morphological Profiles of RNAi-Induced Gene Knockdown Are Highly Reproducible but Dominated by Seed Effects
Source: PLoS One. 2015 Jul 21;10(7):e0131370. doi: 10.1371/journal.pone.0131370 (PMC4511418; doi:10.1371/journal.pone.0131370)

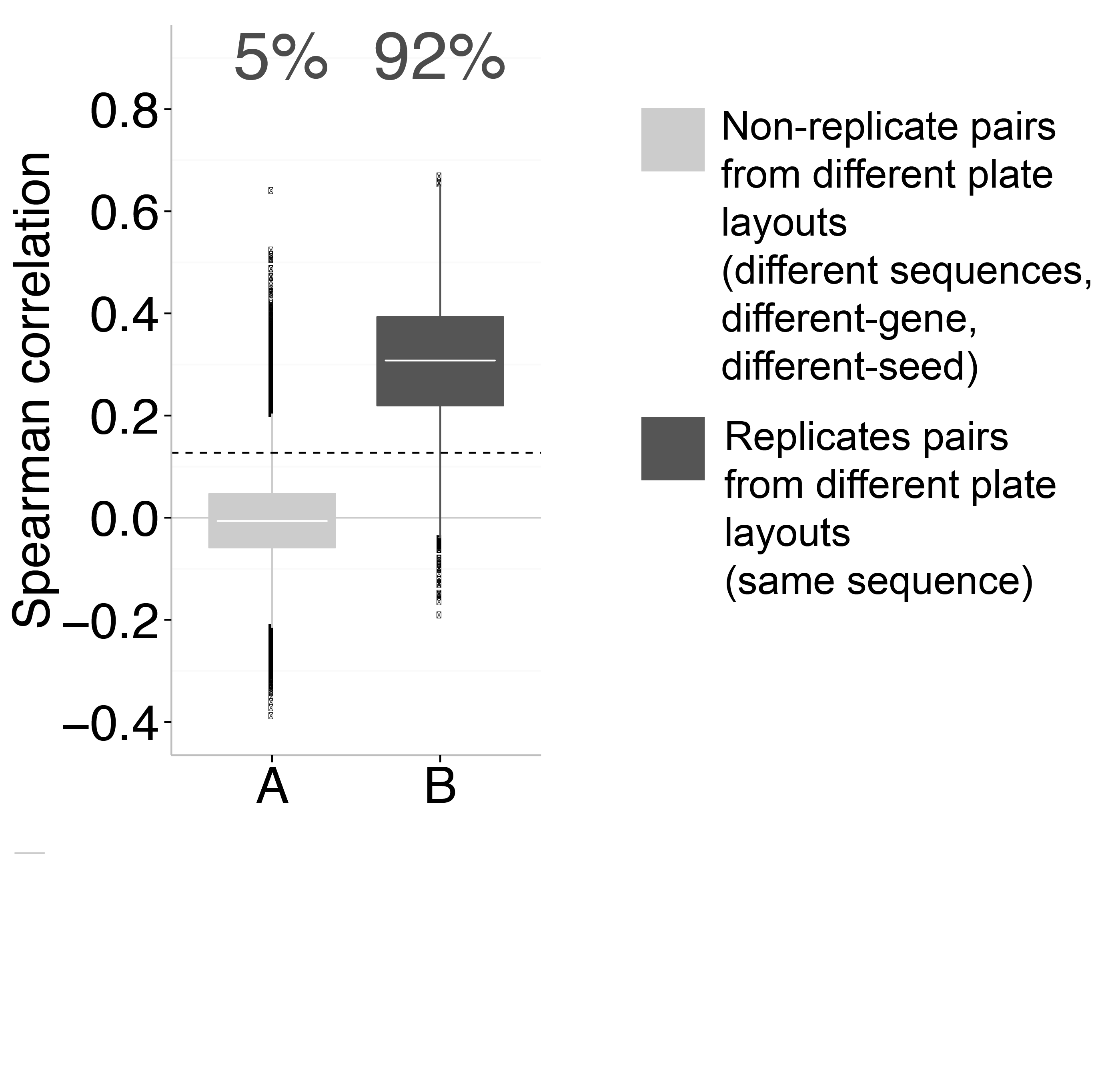

Supplement: S1 Fig — Treatment replicates coming from different well positions (across plates with different layouts) were compared. Using the 95th percentile of the null distribution (A) as a threshold to define significant correlations, 92% of the replicate correlations in B are seen to be significant. Correlations are computed between profiles of individual wells. The percentage of correlations above the defined threshold is indicated; dotted line indicates 95th percentile of the null distribution (A). The difference between means of A and B is highly significant (P-value < 10−15; two-sided Student's t-test). (TIF) [file pone.0131370.s009.tif]

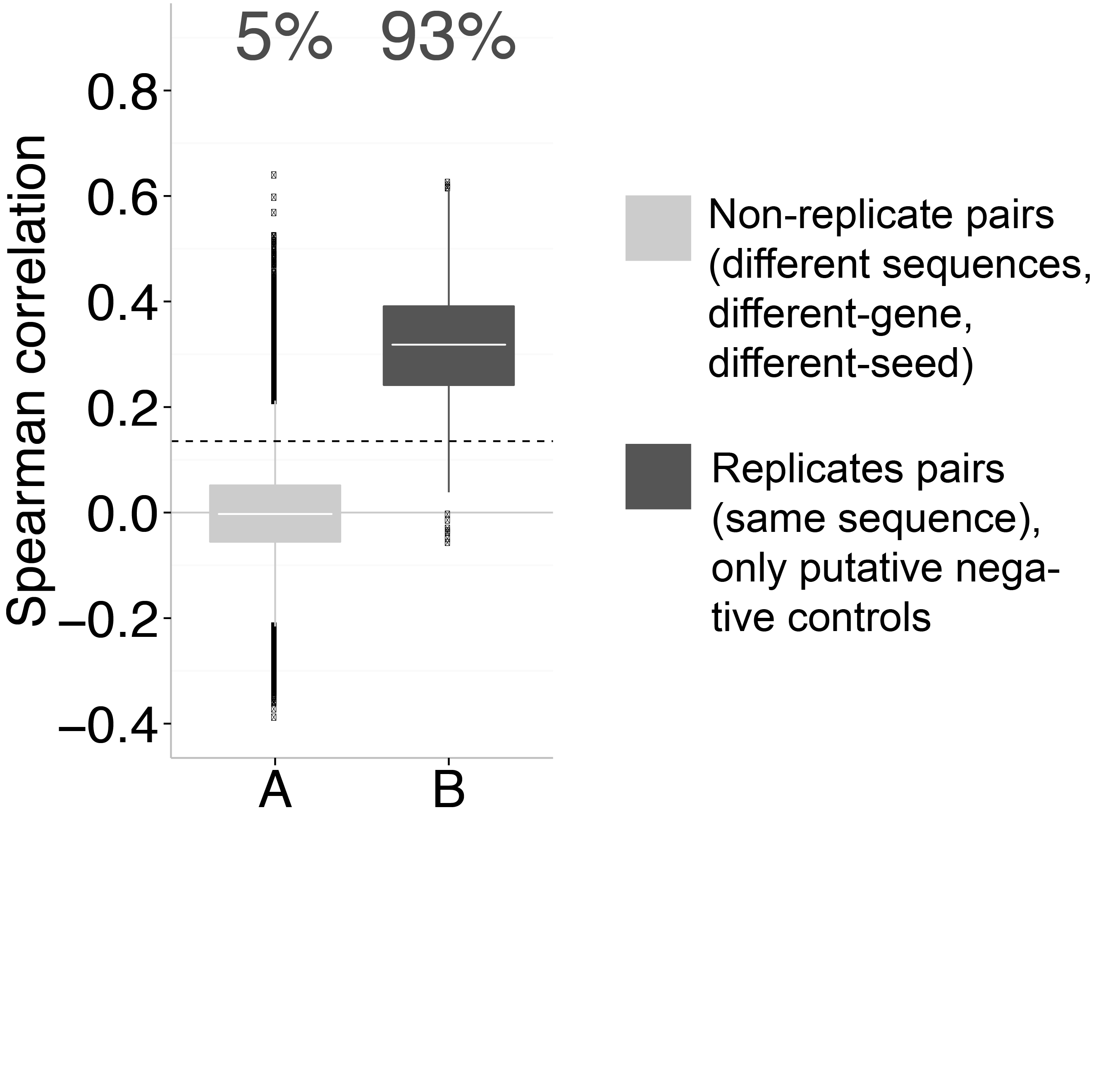

Supplement: S2 Fig — RNAi reagents with sequences not matching any genes in the cell’s genome—thereby putative negative controls—were analyzed for the reproducibility of their image-based profiles. Specifically, shRNA sequences against GFP, LacZ, Luciferase and RFP were considered. 93% of the replicate pairs of these treatments were significantly correlated. Correlations are computed between profiles of individual wells. The percentage of correlations above the defined threshold is indicated; dotted line indicates 95th percentile of the null distribution (A). The difference between means of A and B is highly significant (P-value < 10−15; two-sided Student's t-test). (TIF) [file pone.0131370.s010.tif]

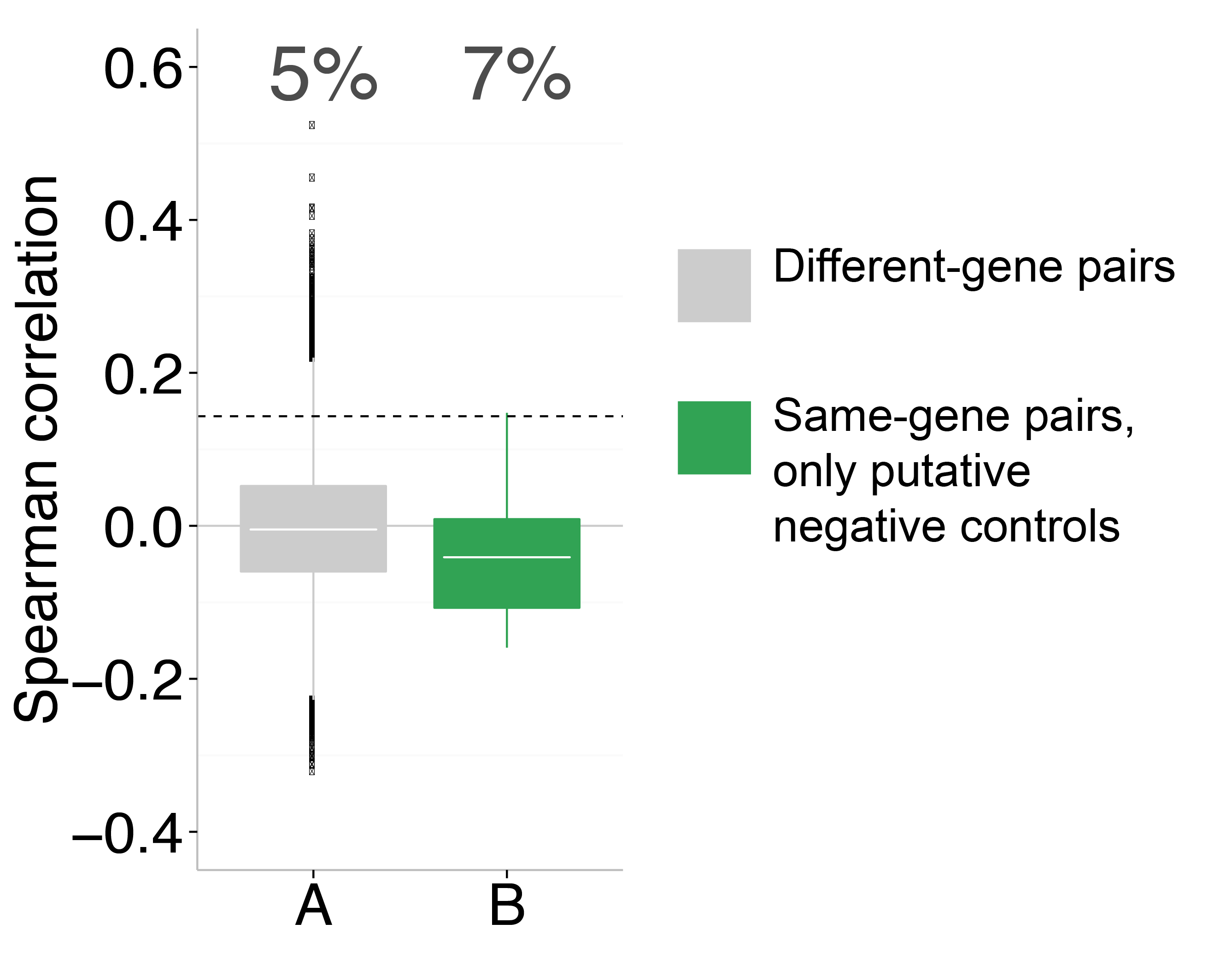

Supplement: S3 Fig — For the putative negative controls considered in S2 Fig, shRNAs sequences targeting the same (non-existent) genes had very low correlation (B). Data was insufficient to do a seed analysis similar to Fig 3C; however the off-target effect is likely to be due to the seed effect. Correlations are computed between profiles of sequences, obtained by median-averaging profiles of replicate wells. The percentage of correlations above the defined threshold is indicated; dotted line indicates 95th percentile of the null distribution (A). (TIF) [file pone.0131370.s011.tif]
