# Supplementary material for: Morphological Profiles of RNAi-Induced Gene Knockdown Are Highly Reproducible but Dominated by Seed Effects
Source: PLoS One. 2015 Jul 21;10(7):e0131370. doi: 10.1371/journal.pone.0131370 (PMC4511418; doi:10.1371/journal.pone.0131370)
Supplement: S1 Text — (DOC) [file pone.0131370.s012.doc]

# Text S1. Data and software

## Software

The image analysis pipelines can be loaded and run using CellProfiler 2.1.0 available at [http://www.cellprofiler.org](http://www.cellprofiler.org/)

The data analysis scripts require the following software

- CellProfiler Analyst (http://github.com/CellProﬁler/CellProﬁler-Analyst/). Clone repository using git clone https://github.com/CellProfiler/CellProfiler-Analyst.git. Add the CellProfiler-Analyst folder to the PYTHONPATH variable.
- R 2.15. Missing R libraries can be installed on the R prompt using install.packages. E.g. install.packages(“plyr”)
- Python 2.7.2. Unless otherwise noted, all python dependencies are available through the Python Package Index (PyPi, http://pypi.python.org/pypi/)

## Image analysis and feature extraction

S3 Dataset contains the morphological profiles for each well in the experiment; this section outlines the instructions to create this dataset from the raw images.

1. **Download raw images.**
   1. Download the BBBC025 Dataset from <https://www.broadinstitute.org/bbbc/BBBC025/> and extract the files.
   2. Download the S7 Dataset and extract the file TAK004_TAK005_U2OS_96H.csv
2. **Download CellProfiler pipelines.**
   1. Download S1 Dataset and extract the files.
   2. Verify that the contents of the folder are
      - analysis.cppipe
      - illum.cppipe
      - isBlurryRules.txt
      - isSaturatedRules.txt
3. **Download source code.**
   1. Download S4 Dataset and extract the files.
   2. Verify that the contents of the folder are

└─ analysis

├─ code

│ ├─ 1-create_cache.py

│ ├─ 2-create_normalizations.py

│ ├─ 3-create_profiles.sh

│ ├─ 4-qc.py

│ ├─ analysis.R

│ ├─ funcs.R

│ └─ profile.data.R

└─ input

└─ TA-MHP-B1

└─ well-summary-profile_mean-median-robust_std-untreated_norm.yml

1. **Generate illumination correction functions (ICFs).** The ICFs are available in S2 Dataset; this step outlines the instructions to create this dataset.
   1. In CellProfiler, load illum.cppipe by selecting File > Import > Pipeline from file.
   2. In CellProfiler, if not already selected, click on the Images module (Input modules > Images) to select it.
   3. Select the folder(s) containing the images from BBBC025 Dataset, and then drag-and-drop the folder(s) into the File list panel, indicated by the words “Drop files and folders here”. The panel will update with the locations of the individual image files.
   4. Select the desired output folder for the illumination functions (View output settings > Default Output Folder).
   5. Run the pipeline to produce ICFs.
2. **Extract image features.**
   1. Unzip contents of S2 Dataset unless the ICFs were generated using step 4, in which case they are in the location specified in Step 4d.
   2. In CellProfiler, load analysis.cppipe by selecting File > Import > Pipeline from file. Change “Metadata file location” (Input module > Metadata) to point to the location of TAK004_TAK005_U2OS_96H.csv.
   3. In CellProfiler, if not already selected, click on the Images module (Input modules > Images) to select it.
   4. Select the folder(s) containing the images from BBBC025 Dataset, and then drag-and-drop the folder(s) into the File list panel, indicated by the words “Drop files and folders here”. The panel will update with the locations of the individual image files.
   5. Select the folder containing the illumination functions (either unzipped from S2 Dataset or generated using Step 4), and drag-and-drop the folder into the File list panel. The panel will add the locations of the illumination functions to the those of the image files.
   6. Update the Rules file location (Analysis modules > Flag image) as follows. For the flag name QCFlag_isSaturated, select “Elsewhere...” for the “Rules file location” setting. In the resulting dialog box, browse to the location of the file isSaturatedRules.txt (located with S1 Dataset). Do the same for the flag name QCFlag_isBlurry, where you will browse to the location of isBlurryRules.txt.
   7. Create a new MySQL database named 2011_07_13_TargetAccelerator for the output and update the Database host, Username, and Password in the ExportToDatabase Analysis module.
   8. Select the desired output folder (View output settings > Default Output Folder).
   9. Run the pipeline to produce the features.
3. **Create per well profiles.**
   1. Copy the .properties file produced in the previous step (in the default output folder) to DatasetS4/analysis/input. Edit the line defining group_SQL_Well by adding the italicized fields as indicated below
      - group_SQL_Well = SELECT ImageNumber, Image_Metadata_Plate, Image_Metadata_Well, *Image_Metadata_6merSeed_12to17, Image_Metadata_7merSeed_11to17, Image_Metadata_ASSAY_WELL_ROLE, Image_Metadata_GeneSymbol, Image_Metadata_IE, Image_Metadata_Number_of_shRNA, Image_Metadata_Same6merSeed, Image_Metadata_Same6merSeedGroup, Image_Metadata_Same7merSeed, Image_Metadata_Same7merSeedPair, Image_Metadata_SameTargetSeq, Image_Metadata_SameTargetSeqPair, Image_Metadata_Target_Sequence, Image_Metadata_shRNA_CloneID* FROM MultipleHairpin_2013_03_07_Analysis_Per_Image
   2. Get the appropriate version of CellProfiler Analyst
      - cd <CellProfiler-Analyst folder>
      - git checkout dce8f51229b8a1d12bc79979b8fa210e00237139
   3. Create local cache of database:
      - cd DatasetS4/analysis/code
      - ./1-create_cache.py TA-MHP-B1
      - Follow the instructions displayed
   4. Create feature normalizations:
      - ./2-create_normalizations.py TA-MHP-B1
   5. Create morphological profiles:
      - ./3-create_profiles.sh TA-MHP-B1
   6. Create the QC file:
      - ./4-qc.py TA-MHP-B1

These steps produce the morphological profiles file well-summary-profile_mean-median-robust_std-untreated_norm.csv and the QC file qc.csv in the folder DatasetS4/analysis/input/TA-MHP-B1/

## Data analysis

The previous section described how to generate the contents of S3 Dataset; this section outlines the instructions to analyze this data.

1. **Download morphological profiles**
   1. Download S3 Dataset, extract the files and copy to DatasetS4/analysis/input/TA-MHP-B1/ (skip if previous section was used to generate the contents of S3 Dataset)
   2. Download S8 Dataset, extract the files and copy clone_search_hairpins.csv to DatasetS4/analysis/input/TA-MHP-B1/
   3. Verify that the contents of DatasetS4/analysis/input/TA-MHP-B1/ are
      - 2011_07_13_TargetAccelerator_MultipleHairpin_2013_03_07_Analysis.properties
      - qc.csv
      - well-summary-profile_mean-median-robust_std-untreated_norm.csv
      - clone_search_hairpins.csv
2. **Download source code** (skip if already extracted in previous section)
   1. As described in Step 3 in *Image analysis and feature extraction*
3. **Run analysis**
   1. In R, setwd to the folder DatasetS4/analysis/code
   2. Execute analysis.R to generate figures.
